# Saudi Medical Students’ Perspectives on the Radiology Curriculum

**DOI:** 10.7759/cureus.69259

**Published:** 2024-09-12

**Authors:** Hussam S Almuhaysin, Johara K Almulhim, Ibrahim Alibrahim, Ali S Alkhars, Ahmed I Alnajjad, Baqir J Albin Ahmed, Hussain M Alsaleh, Hussain A Algafly

**Affiliations:** 1 Diagnostic Radiology, King Faisal University, Al-Ahsa, SAU; 2 Diagnostic Radiology, Imam Abdulrahman Bin Faisal University, Dammam, SAU

**Keywords:** curriculum, medical student, perception, radiology, saudi arabia

## Abstract

Introduction

The importance of radiological technology in healthcare is indisputable in diagnosing and treating many diseases, yet undergraduate radiology education globally remains inadequate. Worldwide, students express dissatisfaction with radiology education, underscoring the need for improvement in this aspect. Saudi Arabia’s medical system lacks a unified radiology curriculum, necessitating robust curriculum development, which should be addressed by the Council of Deans of Saudi Medical Schools. This study aims to assess Saudi medical students’ perceptions of radiology education.

Methods

This is an observational cross-sectional study conducted in the Kingdom of Saudi Arabia (KSA) among undergraduate medical students from King Faisal University (KFU) and Imam Abdulrahman Bin Faisal University (IAU). The study used an online survey to assess medical students’ perspectives regarding the radiology curriculum, the adequacy of the students’ exposure to various imaging modalities, and their confidence in evaluating radiological images. Subjective parameters were measured using a four-point Likert scale, and the results are presented as percentages of students. The data were analyzed using Statistical Package for Social Sciences (SPSS) software, version 29 (IBM Corp., Armonk, NY).

Results

The current paper encompassed 845 participants, revealing a gender distribution of 56.7% (n = 479) female and 43.3% (n = 366) male. Notably, 39.2% (n = 331) and 29.6% (n = 250) aimed for medical and surgical clinician roles, respectively. By contrast, 27.8% (n = 235) expressed uncertainty about their future plans, and only 3.4% (n = 29) intended to pursue radiology as a future career. In evaluating students’ perspectives on the radiology curriculum, more than half of the students (59.4%, n = 502) stated that the amount of radiology education included in their curriculum was inadequate, while 35.7% (n = 302) stated that it was just right, and only 4.9% (n = 41) stated that it was too much. A substantially larger proportion of KFU students (65.2%, n = 334) perceived their clinical exposure to be inadequate compared to IAU students (50.5%, n = 168). Furthermore, a larger percentage of IAU students (46.8%, n = 156) reported “just right” exposure levels compared to KFU students (28.5%, n = 146). This disparity is statistically significant (p < 0.001). Our study demonstrated that X-ray imaging is the most encountered modality during clinical rotations, with 87.2% (n = 737) of students reporting exposure, followed by CT scans (75.2%, n = 636).

Conclusion

The majority of Saudi medical students in the Eastern Province reported an “inadequate” level of exposure to radiology. By identifying shortcomings and inadequacies in the current system, the research may provide a foundation for future improvements, ensuring that medical graduates in the Eastern Province possess the requisite knowledge for competent and safe clinical practice.

## Introduction

Over the past few decades, radiological technology has made enormous advancements [1]. This rapid progress has made medical imaging an essential component of the healthcare system. Despite its importance, there has been a continuous insufficiency in teaching radiology to undergraduate students globally [2]. In addition, despite these advancements, many medical schools do not require a radiology clerkship; for example, only 16% of US medical schools mandate that students complete a radiology clerkship as part of their medical education [3]. Numerous medical schools are integrating radiology education into broader medical specialties like internal medicine, surgery, or emergency medicine rather than having a standalone radiology clerkship [1]. Therefore, radiologists’ participation in teaching at the undergraduate level is crucial to guarantee that future physicians possess current and pertinent knowledge essential for safe practice. This involvement ensures their capability to prescribe necessary imaging procedures for patients and collaborate effectively with radiologists to enable appropriate management based on the imaging results [4].

The Saudi medical education system is structured as follows: it begins with a preparatory year, followed by two years of fundamental and medical science courses. This is followed by three years of clinical training with rotations in various specialties and concludes with a one-year internship, which is considered the sixth year.

Unlike the United States and the majority of European nations, Saudi Arabia permits recent medical school graduates to practice as general practitioners without the need for residency training [1]. Because general practitioners’ radiology knowledge is theoretically limited to what they were taught in the College of Medicine, a robust radiology curriculum becomes even more essential in treating or referring patients in everyday clinical practice [1]. Several studies have been conducted worldwide to assess medical students’ satisfaction with radiology teaching, in addition to the confidence in their knowledge regarding basic radiological principles. In the United States, 72% of students expressed that they had received an inadequate level of exposure to radiology during medical school [5]. In Egypt, around 75% of undergraduate students expressed that the amount of radiology teaching they received was “too little” [2]. In Kuwait, over half of medical students (55%) perceived the radiology education they received as insufficient or inadequate [6]. Medical students have an almost universal perception of undergraduate radiology education throughout the world, and the word “inadequacy” is recurrent when discussing it [1,2,5,6].

The Eastern Province of Saudi Arabia has not been explored extensively in this area. It has the third largest population in the Kingdom of Saudi Arabia, with a growing population and healthcare needs. This study intends to discover Saudi medical students’ perceptions toward the radiology curriculum and pinpoint any shortcomings or inadequacies in its delivery in the Eastern Province’s universities.

## Materials and methods

Study design and population

A cross-sectional, observational descriptive study was conducted from June 1 to June 23, 2023, among Saudi medical students and interns in the Eastern Province of Saudi Arabia.

The sample size was initially determined using the Raosoft sample size calculator (Raosoft Inc., Seattle, USA) with a 5% margin of error and a 95% confidence level, resulting in 385 participants from King Faisal University (KFU) (population size: 1,893) and 301 participants from Imam Abdulrahman Bin Faisal University (IAU) (population size: 1,372). However, to increase reliability, we collected data from 512 participants at KFU and 333 participants at IAU. The study targeted all undergraduate Saudi medical students from the first year to the sixth year (interns) at KFU and IAU, two public governmental colleges. Preparatory-year students, postgraduate medical students, private medical colleges, and medical students from other parts of Saudi Arabia were excluded. In addition, we categorized the first and second years of medical school as preclinical and the third through sixth years as clinical.

Questionnaire development and data collection

The study used a questionnaire adapted from previous research and underwent a rigorous review by a radiologist and an assistant professor to enhance clarity and reduce bias (see Appendix for the questionnaire) [5]. The questionnaire was distributed via a Google Forms survey (Google LLC, Mountain View, California, USA) on social media. It aimed to evaluate medical students’ perspectives on the radiology curriculum, their exposure to different imaging techniques, their confidence in interpreting radiological images, and to assess various radiology teaching methods. Prior to data collection, informed consent was obtained from every participant.

Statistical analysis

A comprehensive statistical analysis was conducted on the collected data using descriptive and inferential methodologies. Descriptive statistics summarized the participants’ demographic characteristics, such as age and gender. For inferential analysis, a chi-square test was performed to examine differences between categorical groups. Subjective parameters were assessed using a four-point Likert scale, with results presented as percentages of participants. Statistical significance was determined by a p-value of less than 0.05, with a 95% confidence interval. All statistical analyses were conducted using IBM Statistical Package for Social Sciences (SPSS) software, version 29 (IBM Corp., Armonk, NY).

## Results

The present study included 845 participants (Table 1). The gender distribution was 56.7% (n = 479) female and 43.3% (n = 366) male. The year of study distribution was 11.4% (n = 96) in the first year, 12.4% (n = 105) in the second year, 15.4% (n = 130) in the third year, 20.0% (n = 169) in the fourth year, 20.2% (n = 171) in the fifth year, and 20.6% (n = 174) in the sixth year (interns). The participants were divided between IAU (39.4%, n = 333) and KFU (60.6%, n = 512). Regarding future plans, 39.2% (n = 331) aimed for clinical roles, 29.6% (n = 250) aimed for surgical fields, 27.8% (n = 235) were undecided, and 3.4% (n = 29) intended to pursue radiology.

**Table 1:** Sociodemographic and other parameters of all the participants (n = 845) The data are represented as frequency (N) and percentage (%).

		N (%)
Gender	Female	479 (56.7)
	Male	366 (43.3)
Year of Medical School	1st-year medical student	96 (11.4)
	2nd-year medical student	105 (12.4)
	3rd-year medical student	130 (15.4)
	4th-year medical student	169 (20.0)
	5th-year medical student	171 (20.2)
	6th-year medical student (Internship)	174 (20.6)
University	Imam Abdulrahman Bin Faisal University	333 (39.4)
	King Faisal University	512 (60.6)
Future plans	Undecided	235 (27.8)
	Clinician in the medical field	331 (39.2)
	Clinician in the surgical field	250 (29.6)
	Radiology	29 (3.4)

Table 2 shows a comparison between IAU and KFU regarding radiological education and clinical exposure in the Eastern Province. At IAU, 50.5% (n = 168) of the respondents felt that they received too little exposure, 46.8% (n = 156) considered it just right, and 2.7% (n = 9) felt that they had too much exposure. By contrast, at KFU, 65.2% (n = 334) perceived the exposure as too little, 28.5% (n = 146) as just right, and 6.3% (n = 32) as too much. The differences between the universities were statistically significant (p < 0.001), as determined by the chi-square test.

**Table 2:** Difference between the schools in the Eastern Province in terms of radiological education and clinical exposure The data are represented as frequency (N), percentage (%), and the corresponding chi-square value. (*) p-value <0.05 is considered statistically significant.

	Imam Abdulrahman Bin Faisal University (N, %)	King Faisal University (N, %)	Chi-square value	p-value
Too little	168 (50.5%)	334 (65.2%)	17.679	<0.001
Just right	156 (46.8%)	146 (28.5%)	28.729	<0.001
Too much	9 (2.7%)	32 (6.3%)	4.758	0.029

Table 3 compares radiology education between IAU and KFU across several dimensions. In terms of medical school year, 71.2% of IAU students were in clinical years, whereas at KFU, 79.5% were in clinical years, showing a slight difference in distribution. KFU students demonstrated greater participation in clinical radiology training through required rotations (34.0% at KFU vs. 24.9% at IAU) and were more likely to be unsure about their training status (23.1% at KFU vs. 17.1% at IAU). On the other hand, a larger portion of IAU students had not yet commenced clinical training (57.9% at IAU vs. 43.0% at KFU).

**Table 3:** Content and different features of radiology education at Imam Abdulrahman Bin Faisal University (n = 333) and King Faisal University (n = 512) The data are represented as frequency (N), percentage (%), and the corresponding chi-square value. (*) p-value <0.05 is considered statistically significant.

		University		Chi-square value	p-value
		Imam Abdulrahman bin Faisal University	King Faisal University		
		N (%)			
What year of medical school are you currently in?	Preclinical	96 (28.8%)	105 (20.5%)	0.402	0.525
	Clinical	237 (71.2%)	407 (79.5%)	44.875	<0.001
What is your clinical radiology training as a medical student?	Have not entered clinical training	193 (57.9%)	220 (43.0%)	1.765	0.183
	Required rotation	83 (24.9%)	174 (34.0%)	32.221	<0.001
	Unsure	57 (17.1%)	118 (23.1%)	21.262	<0.001
Do preclinical exams include radiology imaging?	No	95 (28.5%)	245 (47.9%)	66.176	<0.001
	Yes	238 (71.5%)	267 (52.1%)	1.665	0.196
Who taught you radiology education?	Non-radiologist	109 (32.7%)	180 (35.2%)	17.442	<0.001
	Radiologist	96 (28.8%)	124 (24.2%)	3.563	0.059
	Not sure	116 (34.8%)	128 (25.0%)	0.590	0.442
	Was not taught	12 (3.6%)	80 (15.6%)	50.260	<0.001
Do schools provide the resources to review radiology images on their own?	No	113 (33.9%)	199 (38.9%)	23.705	<0.001
	Not Sure	108 (32.4%)	148 (28.9%)	6.25	0.012
	Yes	112 (33.6%)	165 (32.2%)	10.140	0.001
During your clinical rotations, how often did you interact with a radiologist?	Never	63 (18.9%)	122 (23.8%)	18.816	<0.001
	Once/twice during year	59 (17.7%)	112 (21.9%)	16.426	<0.001
	Few times a month	42 (12.6%)	54 (10.5%)	1.5	0.220
	Few times a week	39 (11.7%)	53 (10.4%)	2.130	0.144
	Daily	2 (0.6%)	45 (8.8%)	39.340	<0.001
	Not applicable	128 (38.4%)	126 (24.6%)	0.015	0.900

Radiology is included in preclinical exams for 71.5% of IAU students, significantly higher than the 52.1% at KFU. Both universities had a similar proportion of non-radiologists teaching radiology, but IAU had more students reporting instruction by radiologists (28.8% at IAU vs. 24.2% at KFU) and fewer stating they were not taught at all (3.6% at IAU vs. 15.6% at KFU). Regarding resources for reviewing radiology images, both institutions had comparable proportions of students affirming the availability of such resources (33.6% at IAU vs. 32.2% at KFU), with a slightly higher percentage of KFU students being unsure about resource availability. Interaction with radiologists during clinical rotations was more frequent at KFU, where 8.8% reported daily interactions compared to just 0.6% at IAU. However, a substantial portion of IAU students (38.4%) found the question not applicable, suggesting they did not experience relevant clinical rotations, compared to 24.6% at KFU.

**Figure 1:**
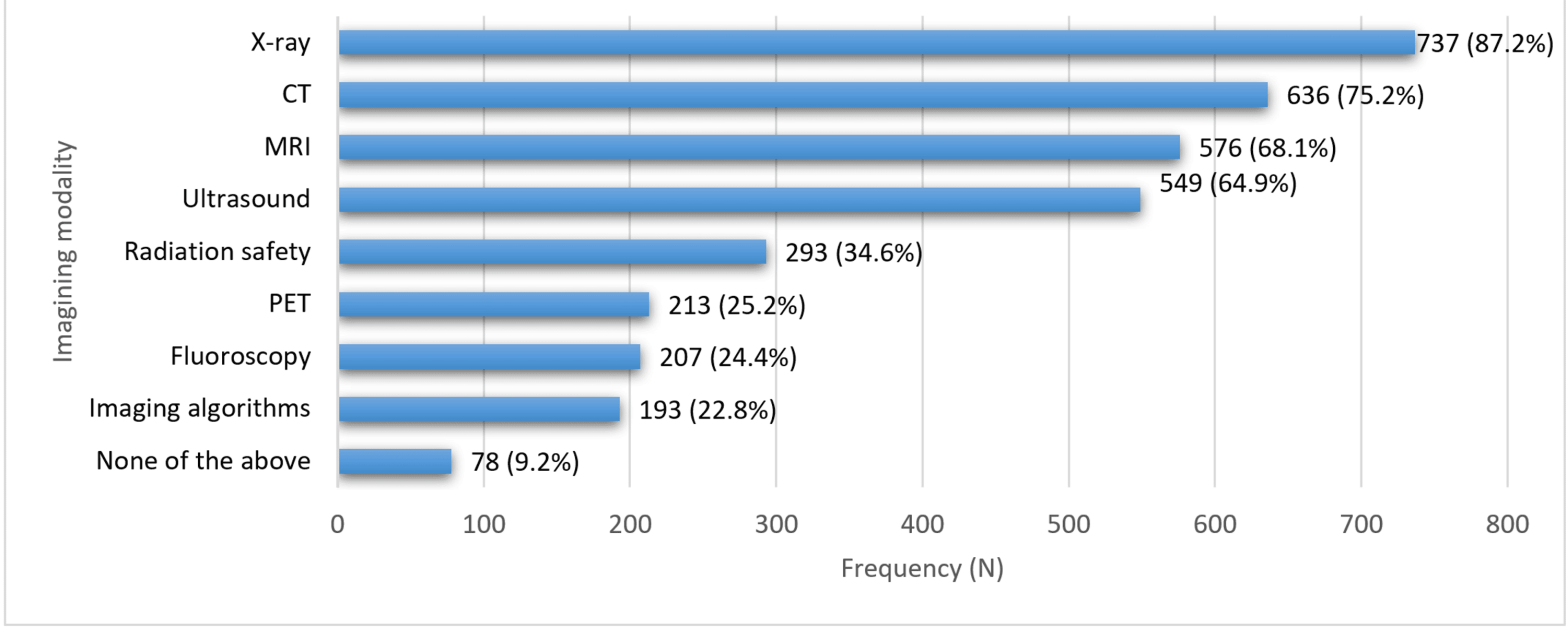
Students exposed to various imaging modalities during their clinical rotations The data are represented as frequency (N) and percentage (%). X-ray: X-radiation, CT: computed tomography, MRI: magnetic resonance imaging, PET: positron emission tomography

Figure 1 shows the exposure of both preclinical and clinical students to various imaging modalities during their clinical rotations. X-ray was the most commonly encountered, with 737 students (87.2%) reporting exposure, followed by CT scans at 636 students (75.2%) and MRI at 576 students (68.1%). Ultrasound was also prevalent, with 549 students (64.9%) having exposure. However, fewer students reported exposure to radiation safety (293 students, 34.6%), PET scans (213 students, 25.2%), and fluoroscopy (207 students, 24.4%). Even fewer students reported exposure to imaging algorithms (193 students, 22.8%). A small percentage of students (78 students, 9.2%) reported not being exposed to any of the listed imaging modalities during their clinical rotations.

**Figure 2:**
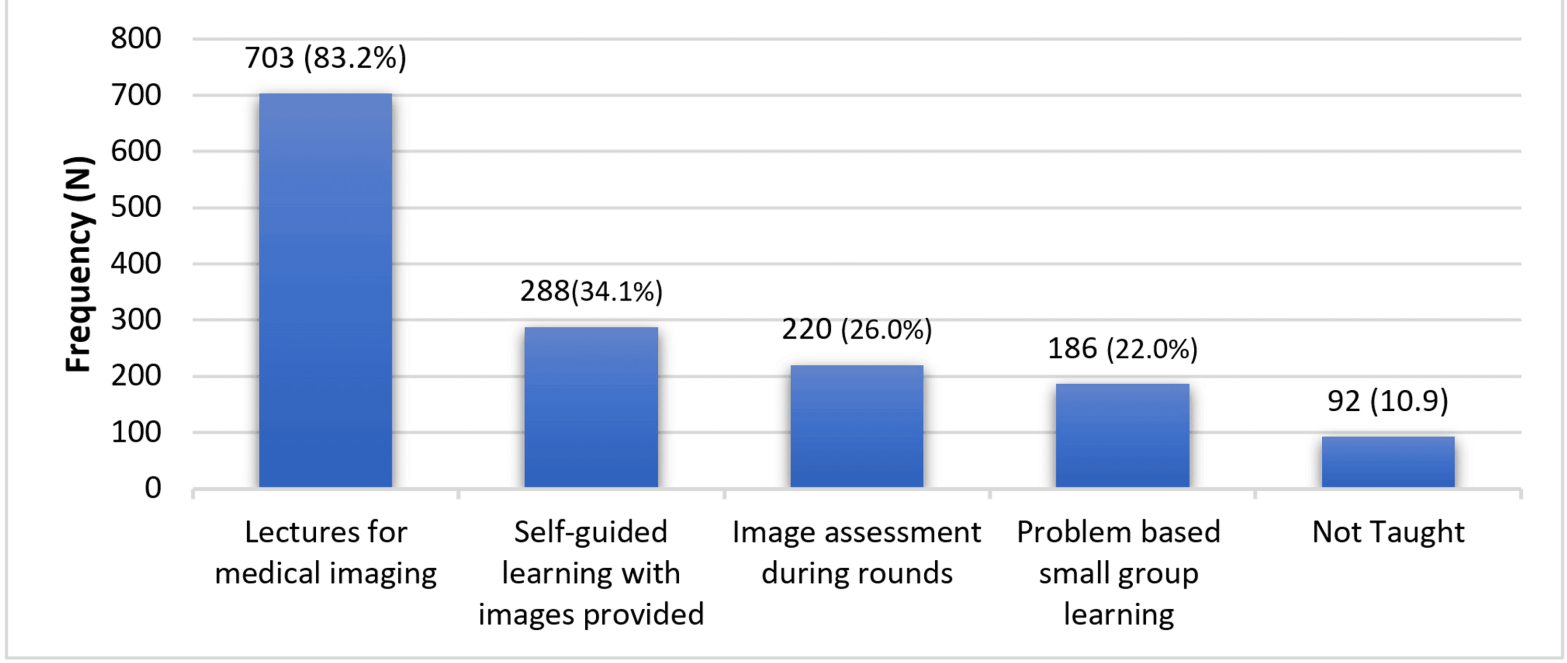
Common teaching methods of imaging among medical schools (n = 845) The data are represented as frequency (N) and percentage (%).

Figure 2 shows the common teaching methods of imaging among medical students. The most prevalent teaching method reported was lectures for medical imaging (n = 703, 83.2%). Following that, self-guided learning with provided images was utilized (n = 288, 34.1%). Image assessment during rounds was reported by 220 respondents (26.0%) as a common teaching method. Problem-based small-group learning was also identified, with 186 respondents (22.0%) reporting its use.

**Figure 3:**
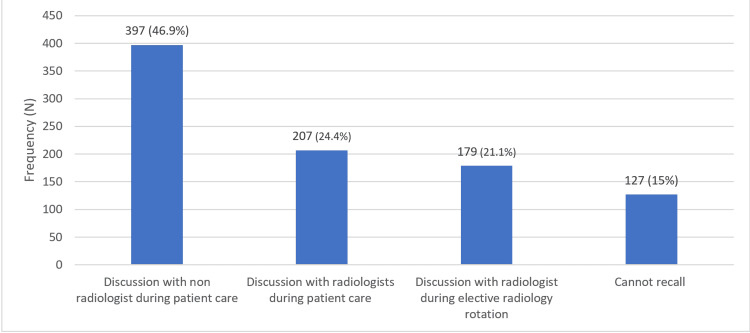
Time at which students encountered or received radiology education The data are represented as frequency (N) and percentage (%).

Figure 3 shows the times when students encountered or received radiology education. The most common time reported was during rounds while discussing with a non-radiologist during patient care, with 397 respondents (46.9%) indicating this timing. Following this, a discussion with a radiologist during patient care was reported by 207 respondents (24.4%). Discussion with a radiologist during an elective radiology rotation was cited as the timing for encountering radiology education by 179 respondents (21.2%). Lastly, 127 respondents (15.0%) could not recall.

**Figure 4:**
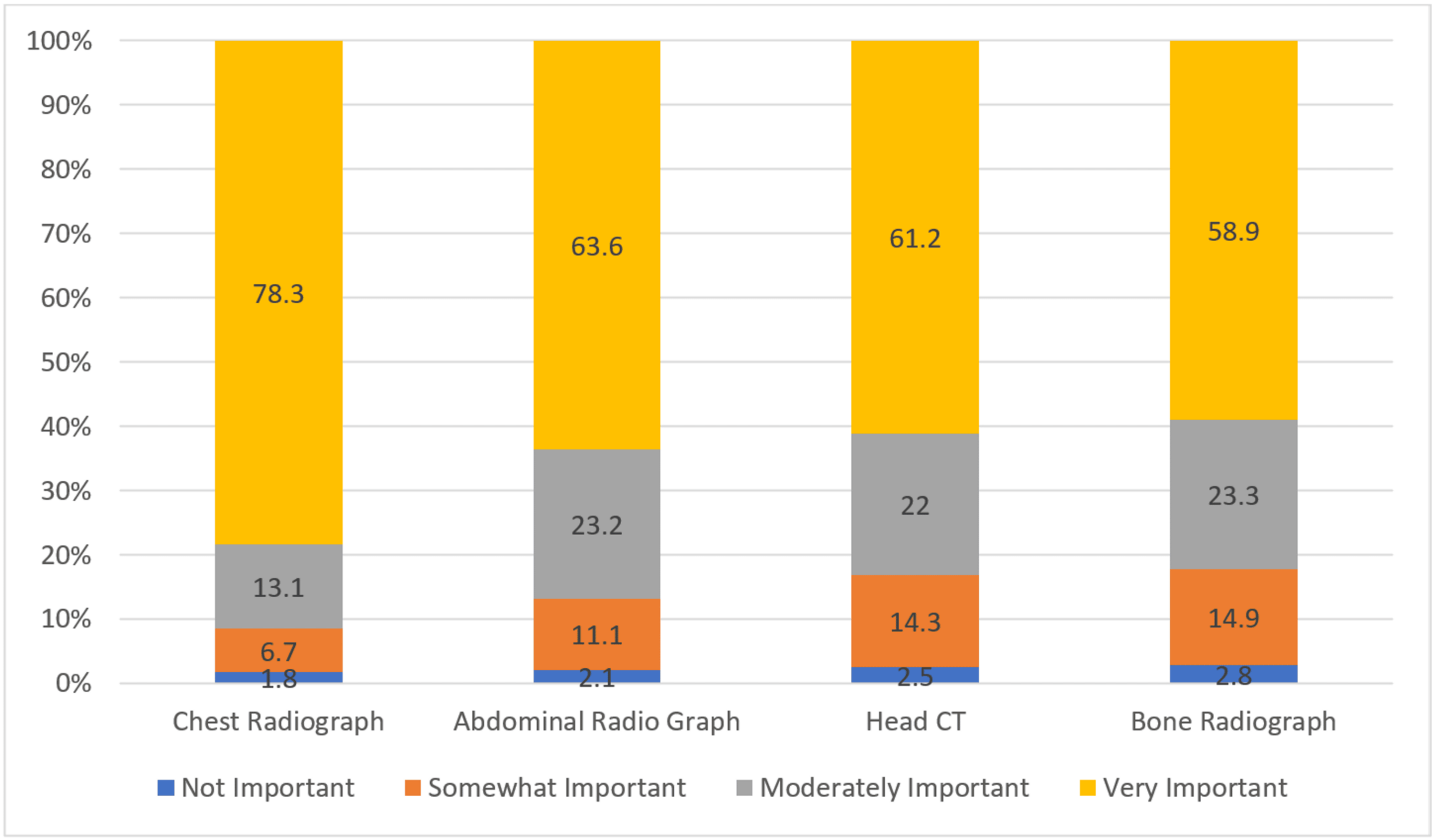
Importance of different systems’ evaluation by radiological images The data are represented as percentage (%).

Figure 4 shows the importance attributed to evaluating different systems through radiological images. Chest radiographs were considered very important by 78.3% (n = 662) of the respondents, while 13.1% (n = 111) found them moderately important. Abdominal radiographs were deemed very important by 63.6% (n = 537) and moderately important by 23.2% (n = 196). Head CT scans were rated very important by 61.2% (n = 517) and moderately important by 22.0% (n = 186). Bone radiographs were considered very important by 58.9% (n = 498) and moderately important by 23.3% (n = 197). Across all modalities, the majority found them either very important or moderately important. The mean values ranged from 3.38 to 3.68, with chest radiographs receiving the highest mean importance rating.

**Figure 5:**
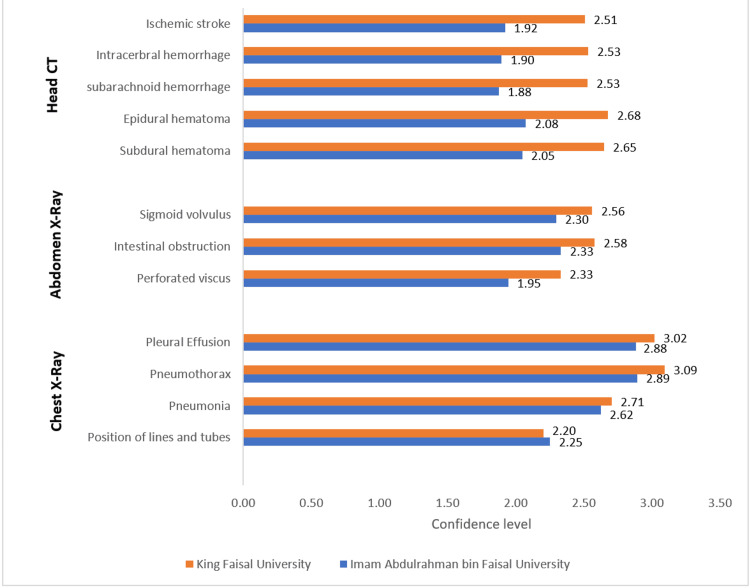
Difference of confidence level in evaluating different pathologies by King Faisal University and Imam Abdulrahman Bin Faisal University students Using four-point Likert scale

As shown in Figure 5, IAU students reported mean confidence scores of 2.25 for the positioning of lines and tubes, 2.62 for pneumonia, 2.89 for pneumothorax, and 2.88 for pleural effusion. Conversely, KFU students exhibited slightly lower mean scores of 2.20 for the positioning of lines and tubes, but higher scores of 2.71 for pneumonia, 3.09 for pneumothorax, and 3.02 for pleural effusion, indicating generally higher confidence levels in identifying pneumonia, pneumothorax, and pleural effusion. For abdominal radiographs, IAU students scored 1.95 for perforated viscus, 2.33 for intestinal obstruction, and 2.30 for sigmoid volvulus, while KFU students scored higher with means of 2.33, 2.58, and 2.56, respectively. In interpreting head CT scans, IAU students rated their confidence at 2.05 for subdural hematoma, 2.08 for epidural hematoma, 1.88 for subarachnoid hemorrhage, 1.90 for intracerebral hemorrhage, and 1.92 for ischemic stroke. By contrast, KFU students reported higher mean scores of 2.65, 2.68, 2.53, 2.53, and 2.51 for these respective conditions.

**Figure 6:**
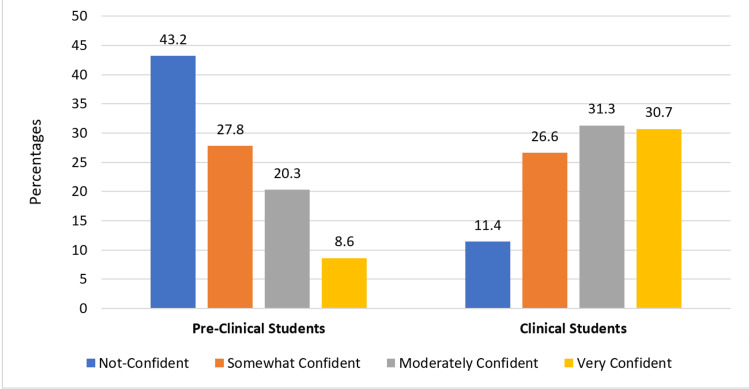
Overall confidence level in evaluating pathologies by clinical and preclinical students (p < 0.001 by a chi-square test) The data are represented as percentage (%). A p-value <0.05 is considered statistically significant.

Figure 6 shows the clinical and preclinical students’ overall confidence levels in evaluating pathologies. Among preclinical students, 43.2% reported not being confident, while 8.6% felt very confident in evaluating pathologies. By contrast, among clinical students, only 11.4% reported not being confident, with 30.7% feeling very confident. The difference in confidence levels between the two groups was statistically significant (p < 0.001), indicating that clinical students generally exhibit higher levels of confidence in evaluating pathologies compared to preclinical students.

## Discussion

Developing diagnostic proficiency among future physicians hinges significantly on the radiology curriculum in medical education. Nonetheless, the adequacy of such education remains a contentious issue. Gunderman et al. suggest that radiology education should be evaluated based on students’ performance relative to their peers, the value it adds to general medicine, and the use of appropriate radiological resources [7]. Meanwhile, Kourdioukova et al. highlight the complexity of defining sufficient radiology education, noting the vast knowledge required and the lack of clarity on its effectiveness from the students’ viewpoint [8]. Studies worldwide reveal widespread dissatisfaction with radiology teaching. Thus, the current paper aims to assess Saudi medical students’ perceptions of the radiology curriculum in the Eastern Province. The study reflects these concerns by revealing substantial disparities in radiology education between IAU and KFU. Our findings shed light on several key aspects of radiology education and its impact on students’ confidence levels, which can inform curriculum development and educational strategies to enhance radiology training.

A significant finding from our study is the perceived inadequacy of radiology exposure among students. A substantially larger proportion of KFU students (65.2%, n = 334) perceived their clinical exposure to be inadequate compared to IAU students (50.5%, n = 168). Furthermore, a larger percentage of IAU students (46.8%, n = 156) reported “just right” exposure levels compared to KFU students (28.5%, n = 146). This difference is statistically significant (p < 0.001). The discrepancy could be attributable to the fact that IAU has a university hospital, whereas KFU does not. Similarly, Chew et al. showed significant differences in radiology assessments across Scottish universities, underscoring the importance of addressing disparities in radiological education [9].

Conversely, KFU students reported higher confidence in diagnosing specific conditions such as pneumonia, pneumothorax, and pleural effusion, exhibiting mean confidence scores of 2.71, 3.09, and 3.02, respectively, compared to IAU students’ scores of 2.62, 2.89, and 2.88. This could suggest that while overall exposure might be perceived as inadequate, the quality of targeted training or contextual learning at KFU may be more effective.

Our study also identified a significant gap in clinical interaction with radiologists. Only 8.8% (n = 45) of KFU students reported daily interactions with radiologists during clinical rotations, compared to just 0.6% (n = 2) at IAU. Enhancing clinical interactions, as suggested by studies, improves diagnostic accuracy and professional confidence among medical students [10].

Moreover, the comparison of radiology education features between preclinical and clinical years highlighted several notable differences. Clinical students reported higher rates of exposure to clinical training, interactions with radiologists, and resources for independent review of radiology images compared to preclinical students. These findings suggest that clinical exposure plays a crucial role in enhancing students’ radiological competence and confidence levels, emphasizing the importance of early integration of radiology training into medical curricula. Tayade et al. show that early clinical exposure in medical education enhances students’ academic strength, clinical skills, confidence, and perception of the specialty, as well as their interest in it as a career [10,11].

Notably, the current paper revealed students’ widespread exposure to various imaging modalities during their clinical rotations, with X-ray (87.2%, n = 737), CT scans (75.2%, n = 636), MRI (68.1%, n = 576), and ultrasound (64.9%, n = 549) being the most commonly encountered modalities. However, fewer students reported exposure to radiation safety (34.6%, n = 293), PET scans (25.2%, n = 213), fluoroscopy (24.4%, n = 207), and imaging algorithms (22.8%, n = 193), indicating potential gaps in exposure to specialized imaging techniques. Efforts to ensure comprehensive exposure to a wide range of imaging modalities are essential to prepare students for diverse clinical scenarios and diagnostic challenges. However, it also poses some challenges that must be addressed to ensure its effective implementation [12].

Probing further, lectures were identified as the most prevalent teaching method for radiology education (83.2%, n = 703), followed by self-guided learning with provided images (34.1%, n = 288), image assessment during rounds (26.0%, n = 220), and problem-based small-group learning (22.2%, n = 186). This highlights the need for a multimodal approach to radiology education, incorporating interactive and case-based learning methods to enhance students’ engagement and comprehension. Similarly, Wachsman et al. showed that clinical case-based teaching combined with interactive web applications improves radiology learning, enhancing students’ ability to identify key imaging pathologies and better preparing them for clinical roles [13].

Overall, clinical students demonstrated higher levels of confidence in evaluating radiological images and interpreting various pathologies compared to preclinical students. However, a significant proportion of both preclinical and clinical students reported varying levels of confidence, indicating the need for ongoing support and training to address knowledge gaps and improve diagnostic skills. Similarly, Burns et al. showed that a one-week radiology boot camp for pre-clerkship medical students enhanced radiology knowledge and skills confidence, potentially addressing the need for expanded radiology content in undergraduate training [14]. Thus, our findings are consistent with prior research, highlighting the significance of clinical exposure, interactive teaching, and a robust curriculum in enhancing radiological competence.

This study has some limitations that should be taken into consideration. First, the observational cross-sectional design limits the ability to establish causality or capture changes over time. Second, while the sample size used in this study meets the criteria for achieving the objectives, using a larger dataset could have improved the reliability of the findings. Third, the study was conducted in only one province, which limits the generalizability of the findings to the other provinces of Saudi Arabia. Finally, the study is based on self-reported data from medical students, which may introduce response bias, as participants might overestimate or underestimate their knowledge and exposure to radiology.

## Conclusions

This paper offers insights into the adequacy of students’ exposure to radiology and students’ perceptions of the radiology curriculum in the Eastern Province of Saudi Arabia. To enhance radiology training and prepare future physicians for the challenges of modern healthcare, it is crucial to address disparities in clinical exposure, integrate interactive teaching methods, and ensure comprehensive curriculum content. By incorporating these findings into educational strategies, medical schools can optimize radiology education and foster the development of competent and confident healthcare professionals.

## Appendices

Study questionnaire

**Table 4:** The questionnaire

Gender:	Male
	Female
What are your future career plans?	Clinician in the medical field
	Clinician in the surgical field
	Radiology
	Undecided
What year of medical school are you currently in?	1st-year medical student
	2nd-year medical student
	3rd-year medical student
	4th-year medical student
	5th-year medical student
	6th-year medical student (Internship)
University:	Imam Abdulrahman bin Faisal University
	King Faisal University
Describe your clinical radiology training as a medical student.	Have not entered clinical training
	Required rotation
	Unsure
Did your preclinical exams include radiology imaging?	YES
	NO
Who primarily taught your medical imaging during preclinical years?	Non-radiologist
	Radiologist
	Not sure
	Was not taught
How did your school teach you medical imaging? (Select all that apply)	Lectures for medical imaging
	Self-guided learning with images provided.
	Problem-based small group learning dedicated to medical imaging.
	Image assessment during rounds
	Not taught
Does your school provide you with resources to go through radiology images on your own?	Yes
	No
	Not sure
In which areas have you received formal education? (select all that apply)	Imaging algorithms
	X-ray
	Radiation safety
	Fluoroscopy
	Ultrasound
	CT
	MRI
	PET
	None of the above
During your clinical rotations, how often did you interact with a radiologist?	Daily
	A few times a week
	A few times a month
	Only once or twice during the year
	Never
	Not applicable
How do you feel about the amount of radiology you have encountered in medical school?	Too much
	Just right
	Too little
During your clinical education, when did you encounter radiology images? (select all that apply)	On rounds while discussing with radiologist
	On rounds while discussing with non-radiologist doctor
	On rounds while discussing with the training doctor
	During a radiology elective
	Not seen
	Not applicable

Rate your confidence level in evaluating the following items on chest radiographs:				
	Not Confident	Somewhat Confident	Moderately Confident	Very Confident
Position of lines and tubes	☐	☐	☐	☐
Pneumonia	☐	☐	☐	☐
Pneumothorax	☐	☐	☐	☐
Pleural Effusion	☐	☐	☐	☐

Rate your confidence level in evaluating the following items on abdomen radiographs:				
	Not Confident	Somewhat Confident	Moderately Confident	Very Confident
Perforated viscus	☐	☐	☐	☐
Intestinal obstruction	☐	☐	☐	☐
sigmoid volvulus	☐	☐	☐	☐

Rate your confidence level in evaluating the following items on head CT:				
	Not Confident	Somewhat Confident	Moderately Confident	Very Confident
Subdural hematoma	☐	☐	☐	☐
Epidural hematoma	☐	☐	☐	☐
Subarachnoid hemorrhage	☐	☐	☐	☐
Intracerbral hemorrhage	☐	☐	☐	☐
Ischemic stroke	☐	☐	☐	☐
